# Hydrogels Based on Imino-Chitosan Amphiphiles as a Matrix for Drug Delivery Systems

**DOI:** 10.3390/polym12112687

**Published:** 2020-11-14

**Authors:** Daniela Ailincai, William Porzio, Luminita Marin

**Affiliations:** 1 “Petru Poni” Institute of Macromolecular Chemistry, 400487 Iasi, Romania; lmarin@icmpp.ro; 2 Institute of Chemical Sciences and Technologies, “G. Natta“ Consiglio Nazionale delle Ricerche (SCI-TEC) via A. Corti, 12 20133 Milano, Italy; william.porzio@scitec.cnr.it

**Keywords:** chitosan, citral, diclofenac sodium salt, drug delivery systems

## Abstract

This paper reports new formulations based on chitosan, citral, and diclofenac sodium salt (DCF). The central idea was to encapsulate an anionic drug into a polycationic hydrogel matrix in order to increase the intermolecular forces between them and thus to ensure slower drug release, while citral was used as a penetration enhancer to assure efficient delivery of the drug. Hydrogels without drug were also synthesized and used as a reference. The structure, morphology, and supramolecular architecture of the drug delivery systems were evaluated by FTIR spectroscopy, scanning electron microscopy, polarized optical microscopy, and wide-angle X-ray diffraction. The drug release kinetics was monitored in vitro by UV-VIS spectroscopy, in physiological conditions, while the enzymatic and hydrolytic degradability of the hydrogels were evaluated in the presence of lysozyme and phosphate buffer saline (PBS), at 37 °C. All of the data revealed that the anionic DCF was strongly anchored into the polycationic matrix and the drug was slowly released over 7 days. Moreover, the release rate can be controlled by simple variation of the molar ratio between the polycationic chitosan and lipophilic citral.

## 1. Introduction

Among biomaterials, hydrogels are one of the most versatile classes in terms of properties and applicability [1,2]. Presenting different morphological and supramolecular peculiarities, hydrogels are used in biomedicine as implants, as a matrix for cell growth or in gene therapy, as platforms for drug delivery, etc. [3,4]. In the field of drug delivery, hydrogels are suitable and have been extensively used for all administration routes, from local to systemic, in oral or transdermal drug delivery [5]. Even if hydrogels are usually able to encapsulate large amounts of drugs, the efficacy of the treatment is not always the highest due to the low penetration ability of the drug through different barriers of the human body. That is why, usually, besides the carrying matrix and the drug itself, the formulations also include different agents, which have the ability to improve the drug’s penetration through the skin etc. [6]. There are many synthetic agents which are used as penetration enhancers, such as solvents, sulfoxides, pyrolidones, azones, and surfactants, but their use in a formulation increases the costs and raises issues related to their safety and biocompatibility [7]. Considering that biocompatibility is a requirement for all of the components of drug delivery systems, hydrogels are formulated based on natural polymers or polymers from renewable resources, which have gained an increasing amount of attention during the last few years [8]. Among this class, chitosan plays an important role, not only due to its tremendous biological properties, but also because it is very similar to the extracellular matrix, from both compositionally and mechanically points of view, which represents an important advantage regarding its bioapplicability [8,9,10,11].

Moreover, the molecules used to induce chitosan gelation must also be biocompatible [12,13]. On this basis, one of the research directions intensely studied has focused on developing a new strategy to crosslink chitosan chains using biologically-friendly monoaldehydes [14,15,16]. This is possible due to the fact that the aldehydes are able to react with chitosan, forming dynamic amphiphilic polyimines, which, due to the imination and trans-imination processes, along with the hydrophobic-hydrophilic segregation, self-assemble in tridimensional polymeric networks, which are actually hydrogels.

Among the systems synthesized in our group, following this novel synthetic approach, hydrogels based on the self-assembling of citryl-imine-chitosan derivatives presented excellent properties for bio-applications, such as biocompatibility and thixotropy [15]. Moreover, in the last few years, it was demonstrated that the use of citral in different drug delivery systems increased the cytoplasmatic absorption of both hydrophilic and hydrophobic drugs, such as termoporfin, ferulic acid, and meloxicam [5,6]. Due to its hydrophobic nature, citral proved to be able to modify the highly ordered structure of the phospholipid layer, increasing its permeability, and thus enhancing the drug’s diffusivity through the skin. Studies related to its metabolization in mice proved that citral is fully and rapidly metabolized after dermal treatment, demonstrating its safety in biomedical applications, and constituting an alternative to synthetic penetration enhancers [5,6].

Therefore, the use of cytril-imine-chitosan hydrogels as a matrix for drug delivery presents multiple advantages, due to the presence of both chitosan and citral. A preliminary investigation of their use as a matrix for drug delivery systems of 5-fluorouracil (5–FU) highlighted the importance of an appropriate choice of the drug in such a matrix; because of the poor anchoring of the drug into the chitosan matrix, it crystallized as big crystals in the hydrogels, prompting a significant burst release and thus decreasing the performances of these systems in drug delivery [17].

In this context, it was decided to make use of the promising properties of citryl-imine-chitosan hydrogels, by using a drug with more suitable structural particularities for developing strong interactions with the polymeric matrix. Therefore, diclofenac sodium salt (**DCF**) was chosen and used as an anionic drug model, due to its appealing chemical structure from the point of view of the interactions which may appear between its molecules and the ones of the chitosan matrix. The strong anionic character of DCF is complementary to the polycationic character of chitosan, promoting electrostatic interactions. Furthermore, the chlorine, nitrogen, oxygen, and amino groups in DCF molecules may form numerous hydrogen bonds with the hydroxyl, amine, and amide groups of chitosan [18,19,20]. All of these physical interactions may bond the drug molecules to the chitosan matrix, retarding drug release. In this manner, novel drug delivery systems with sustained release should be obtained, with performances which will decrease the frequency of dose administration, thus increasing the patient’s compliance [21,22].

## 2. Materials and Methods

### 2.1. Materials

Citral (95%), chitosan (217.74 kDa, DA: 85%), phosphate buffer saline (PBS) (pH = 7.4), diclofenac sodium salt (DCF), lysozyme (lyophilized powder, protein 90%, 40,000 units/mg protein), and ethanol were purchased from Sigma Aldrich (Steinheim, Germany) and used without any purifications.

### 2.2. Synthesis of the Hydrogels and the Corresponding Drug Delivery Systems

**Scheme 1:**
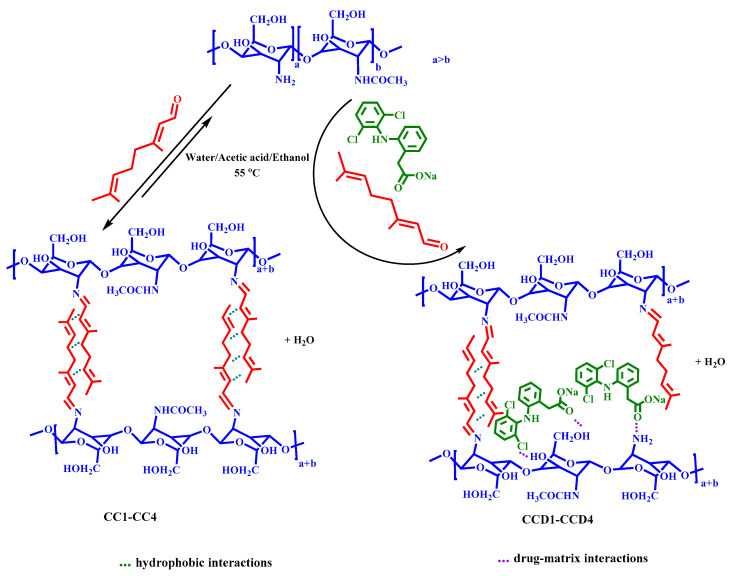
Reaction scheme for the synthesis of the reference hydrogels (**CC1-CC4**) and the drug delivery systems obtained by encapsulating diclofenac sodium salt (**CCD1-CCD4**).

Four drug delivery systems (samples **CCD1-CCD4**) were obtained by the self-assembling of citryl-imino-chitosan amphiphiles in the presence of an anti-inflammatory drug called diclofenac sodium salt (Scheme 1). All of the systems contained the same amount of drug (1.5 mg DCF in 62 mg xerogel), but they differed in terms of the molar ratio between the amino groups of chitosan and the aldehyde group of citral (Table 1). Hydrogels without DCF drug were also obtained and characterized and used as references (samples **CC1-CC4**). The drug delivery systems were coded with **CCD**, followed by a number which represents the molar ratio between the chitosan’s and citral’s functionalities used when obtaining them. For example, the sample **CCD4** was obtained by combining the polymer and monoaldehyde at a molar ratio of 4 to 1, and contained diclofenac sodium salt. The corresponding control sample was **CC4**, which was synthesized by combining the reagents at the same molar ratios, but without encapsulating the **DCF** drug, according to Table 1.

The experimental procedure applied for the in situ preparation of the drug delivery systems was as follows. A 2% solution of chitosan was prepared by dissolving chitosan in 0.7% acetic acid. After chitosan dissolution, the temperature of the system was increased to 55 °C, after which a co-solution of citral and DCF (1%) in ethanol was slowly added under vigorous magnetic stirring. The hydrogelation time increased as the amount of citral decreased: The hydrogelation occurred instantaneously in the case of **CCD1**, in ten minutes for **CCD2**, in 5 h for **CCD3**, and in 24 h for **CCD4**. The hydrogelation in the case of the reference samples occurred faster than in the case of the drug delivery systems.

### 2.3. Methods

ATR-FTIR spectra of the drug delivery systems (**CCD**) and the reference samples (**CC**) were registered on an FTIR Bruker Vertex 70 Spectrophotometer equipped with a ZnSe single reflection ATR accessory. The measurements were performed in the 4000−600 cm^−1^ range, with 32 scans and a resolution of 4 cm^−1^.

Wide angle X-ray diffraction (WAXD) of the samples was performed on a Siemens D-500 equipped with Soller slits (2°) placed before the sample, 0.3° aperture and divergence windows, and a VORTEX detector with an extreme energy resolution specific for thinner films. Cu-Ka radiation (λ = 0.1541 nm) with a power use of 40 kV × 40 mA was adopted, with each diffractogram being recorded with steps of 0.05° (2 theta) and a 6 s measure time. The diffractograms were recorded in the range of 2–45 2 theta degrees, at 25 °C, on xerogel pellets placed on a steel sample holder, which did not interfere with organic materials’ diffraction effects. The pellets were obtained by pressing the xerogels in a hydraulic press with a force of 5 N/m^2^.

The degree of ordering of the samples at a supramolecular level was also investigated by polarized optical microscopy (POM), using a Leica DM 2500 microscope.

The morphological investigation of the samples was done with a field emission scanning electron microscope (Scanning Electron Microscope SEM EDAX–Quanta 200) at an accelerated electron energy of 20 eV. The morphology was investigated on xerogels obtained by freeze-drying. The size of the pores, the pore wall thickness, and the dimensional polydispersity of the pores were measured using the Image J Software.

Atomic force microscopy (AFM) images were recorded with an Ntegra Spectra Instrument (NT-MDT, Russia) in a semicontact mode, at room temperature and using a silicon cantilever (NSG 10) on hydrogel samples deposited on a mica substrate.

The in vitro release kinetics was monitored in PBS with a pH = 7.4. Pellets of drug delivery samples with a mass of 62 mg were immersed in a volume of 10 mL PBS and kept at 37 °C. At certain times, 2 mL of supernatant was removed from the vials and replaced with PBS, maintaining a constant volume of 10 mL [23,24]. The concentration of the drug in the supernatant was determined by UV-Vis spectroscopy, according to Lambert–Beer law, using the maximum of absorbance from 275 nm, and fitting the value on a previously drawn calibration curve. The percentage cumulative DCF release from the samples was calculated according to the following mathematical equation:

% DCF = [(10C_n_ + 2C_n−1_)/m_0_] × 100, (1)

in which C_n_ and C_n−1_ represent the DCF concentrations in the extracted supernatant after n and n − 1 withdrawing steps, respectively, while m_0_ = 1.5 mg represents the amount of DCF loaded in the drug delivery systems. All of the experiments were conducted in triplicate and the values given in the graphs are the mean value of three independent measurements. The absorbance of the released DCF drug was measured using a Perkin Elmer Lambda 35 UV-Vis spectrophotometer.

The release kinetics of the drug was evaluated by fitting in vitro release data to five mathematical models, as follows:

(i) *Zero order model*: $Q_{t}=K_{0}\cdot t$, where Q_t_ is the amount of drug dissolved in the time *t* and K_0_ is the zero order release constant;
(ii) *Higuchi model*: $Q_{t}=K_{H}\cdot t^{\frac{1}{2}}$, where Q_t_ is the amount of drug released in the time *t* and K_H_ is the Higuchi dissolution constant;
(iii) *Hixson*–*Crowell model*: $W_{0}^{1/3}-W_{t}^{1/3}=K\cdot t$, where W_0_ is the initial amount of drug in the formulation, W_t_ is the remaining amount of drug in the formulation at time *t*, and K is a constant;
(iv) *Korsmeyer*–*Peppas model*: $\frac{M_{t}}{M_{\infty }}=K\cdot t^{n}$, where M_t_/M_∞_ is the fraction of drug released at the time *t*, K is the release rate constant, and n is the release exponent;
(v) *First order model*: $logQ_{t}=logQ_{0\;}+K\cdot t/2.303$, where Q_t_ is the amount of drug released in the time *t* and K is the first order release constant.

The in vitro enzymatic degradation of the reference samples was evaluated quantitatively by mass loss determinations of the xerogels immersed and kept in lysozyme buffer solution in comparison with the same amount of xerogel immersed in PBS. Pieces of xerogels of 10 mg each were added to 10 mL of lysozyme solution (10 mg/L) and kept at 37 °C for different periods of time [25]. The lysozyme solution was removed and replaced with freshly prepared solution every three days. At certain time intervals, the pieces were taken out, washed with double distilled water to remove the salts, and submitted to gravimetric measurements and SEM analysis. The mass loss was calculated using Equation (2), as follows:

(2) $$W_{loss}=\frac{W_{0-\;}W_{t}}{W_{0}}\cdot 100,$$

where W_loss_ = the weight loss of the hydrogel, W_0_ = the initial weight of the lyophilized hydrogel, and W_t_ = the weight of the xerogel at a predetermined moment.

## 3. Results and Discussion

A series of four drug delivery systems (**CCD1-CCD4**) was obtained by the self-assembling of citryl-imino-chitosan amphiphiles in the presence of the DCF anti-inflammatory drug. The drug delivery systems contained the same amount of drug, but they differed in terms of the density of citral side chains on the chitosan backbone. Citryl-imino-chitosan hydrogels were proved to be biocompatible and a preliminary investigation of their ability to act as a matrix for 5-FU demonstrated their ability to release in vivo the 5-FU in a sustained manner, assuring its longer bioavailability while diminishing the side effect. This was despite the fact that the 5-FU was encapsulated as large crystals with a faster dissolution rate [17]. These findings encouraged further investigations on this matrix, in order to find the proper profile of the drugs to be encapsulated and take advantage of these materials for real-life bioapplications. Considering the potential of anchoring an anionic drug into the polycationic chitosan matrix, it was decided to use diclofenac sodium salt as the anionic drug model. Moreover, in situ encapsulation of the drug during the hydrogelation process was chosen for formulation preparation, because it may further support fine dispersion into the matrix.

### 3.1. Structural and Morphological Characterization by FTIR Spectroscopy

FTIR spectroscopy was used in order to characterize the obtained drug delivery systems at two different levels, as follows: (i) Structurally—in order to check the formation of the imine linkage between chitosan and citral in the presence of the DCF drug, and (ii) morphologically—to evaluate the changes of the polymeric matrix and the DCF drug due to their mutual influence on each other. Therefore, by comparing the FTIR spectra of the drug delivery systems (**CCD1-CCD4**) to the ones of the reference xerogels (**CC1-CC4**), no important differences could be observed regarding the bands’ position and number, indicating that the gelation mechanism did not change due to the presence of DCF. Our previous studies demonstrated that by reacting chitosan with citral, amphiphilic imines are formed, which, due to the imination and transamination reactions, along with the hydrophilic-hydrophobic segregation, are able to self-assemble into three-dimensional polymeric networks, which are in fact hydrogels [17]. Therefore, by analyzing the FTIR spectra of the drug delivery systems in comparison to the ones of the reference xerogels, it can be concluded that hydrogelation is a consequence of both the imination reaction and the segregation of the formed imines into hydrophobic clusters.

**Figure 1:**
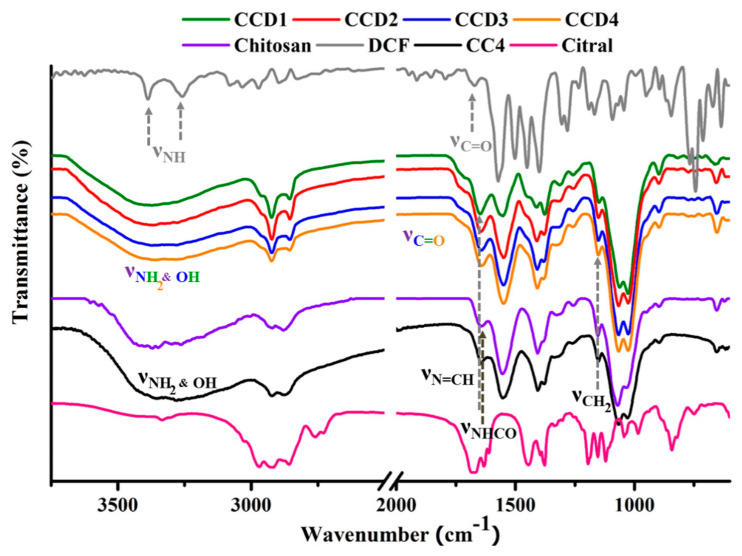
FTIR spectra of chitosan, citral, reference xerogel sample **CC4**, diclofenac sodium salt (**DCF**), and drug delivery systems **CCD1-CCD4**.

Therefore, in the fingerprint region, the band at 1645 cm^−1^ corresponding to the group stretching vibrations of the newly formed imine bonds between chitosan and citral appeared in the spectra of the drug delivery systems, while the vibration of the aldehyde unit of cytral (1675 cm^−1^) disappeared, indicating that the imination process was not impeded by the presence of DCF (Figure 1). Upon a deeper view, it could be observed that the band at 1725 cm^−1^, which is characteristic of the stretching vibrations of the C=O groups in the IR spectrum of the DCF drug, also appeared in the spectra of the drug delivery systems as a shoulder, which was a sign of DCF’s presence in the matrix [26,27]. Moreover, this band was a little bit shifted in the spectra of the drug delivery systems, very probably due to the strong interactions with the matrix.

### 3.2. Supramolecular Characterization by Wide Angle X-ray Diffraction and Polarized Optical Microscopy

**Figure 2:**
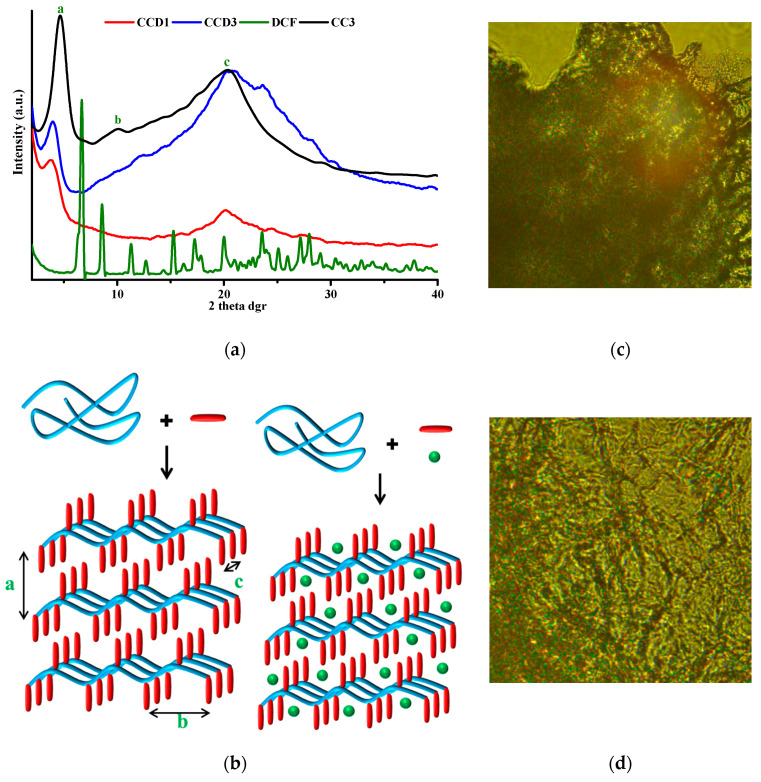
(**a**) Wide angle X-ray diffractograms of the xerogel sample **CC3**, DCF, and drug delivery systems **CCD1** and **CCD3**; (**b**) schematic representation of the supramolecular arrangement of the xerogels and drug delivery systems; and polarized optical microscopy (POM) images for (**c**) a representative reference hydrogel (**CC3)** and (**d**) **CCD3**.

Previous studies revealed that chitosan’s hydrogelation with monoaldehydes, with citral in particular, is possible due to the hydrophobic-hydrophilic segregation of the resulting amphiphilic imino-chitosan derivatives. In order to evaluate the supramolecular architecture of the drug delivery systems and to investigate the physical state in which the DCF drug was encapsulated in the hydrogels, wide angle X-ray diffractograms were produced for the DCF drug, the reference samples, and the drug delivery systems. By comparing the diffractograms of the reference samples to the ones of the drug delivery systems, the occurrence of the two main reflections, characteristic of the architecting of the amphiphilic cytril-imine-chitosan into layered clusters, could be observed indicating that their formation also occurred in the presence of DCF and consequently, the hydrogelation process (Figure 2). However, both reflections significantly decreased in intensity and a slight shift (towards larger d-spacing) of the band corresponding to the interlayer distance was observed in the diffractograms of the drug delivery systems compared to the reference samples. This indicates that the presence of the drug slightly hindered the hydrophilic-hydrophobic segregation. Moreover, by analyzing the diffractograms of the drug delivery systems in comparison to that of the DCF drug, it could be observed that some bands characteristic of the drug were present in the formulations as low broad maxima in the wide-angle domain, while those at a lower angle disappeared. This maps a change in the crystallization pattern of DCF in the hydrogel matrix, with the strong intermolecular forces between two components guiding the growth of two-dimensional arrays [28].

In order to obtain a visual insight into the material ordering, POM was used as a complementary method for an evaluation of the supramolecular architecture of the drug delivery systems. At a first glance, all of the samples presented birefringence under polarized light, with a fine, banded texture similar to the neat hydrogels, with no visible crystals, confirming the absence of micrometric three-dimensional crystals of the drug [17,29].

### 3.3. Morphology at a Micro and Nano Level Observed by SEM and AFM

**Figure 3:**
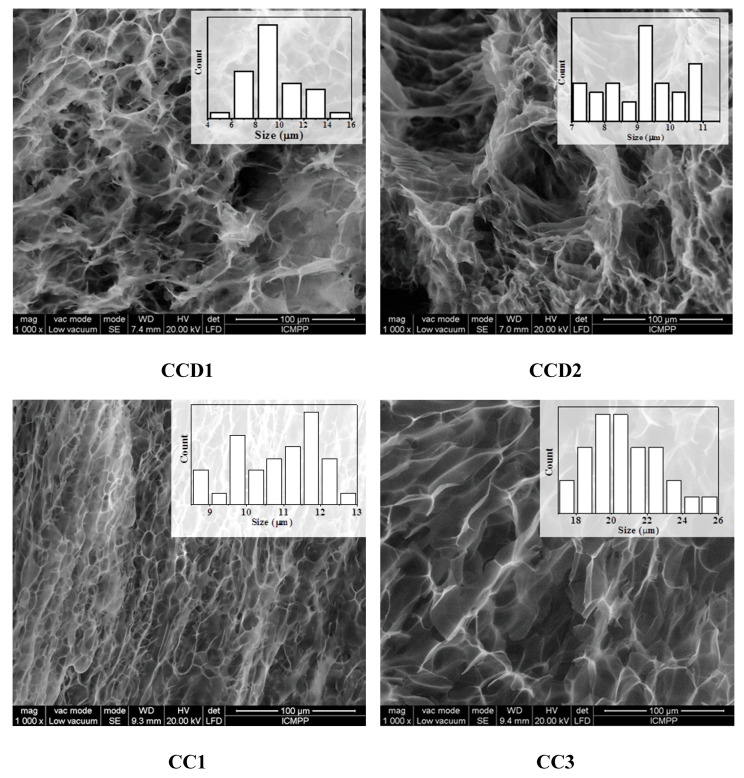

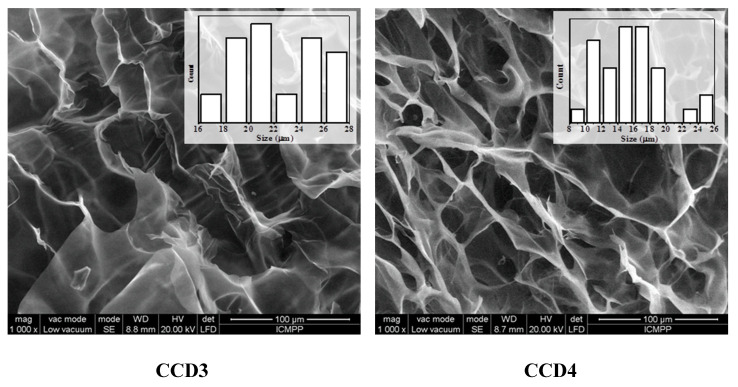
Representative SEM images of the drug delivery systems **CCD1**-**CCD4** and xerogel samples **CC1** and **CC3**.

The morphological investigations using SEM revealed highly porous microstructures in the case of the four drug delivery systems. All of the samples presented micrometric pores with different sizes, depending on the molar ratio between chitosan’s amino group and the aldehyde group of citral. More precisely, the size of the pores increased from 9 μm for the **CCD1** sample to 22 μm in the case of the sample **CCD4** (Figure 3, Table 2). No significant differences could be observed between the morphology of the **CCD1-CCD4** drug delivery systems and that of the **CC1-CC4** reference samples, with the lack of micrometric drug crystals indicating the prevalence of DCF–matrix interactions to the detriment of DCF–DCF interactions—data which are in agreement with the previous findings recorded by X-ray and POM techniques (see Section 3.2).

This, combined with the greater thickness of the pore walls in the case of the drug delivery systems in comparison to those of the reference samples, confirmed the very fine encapsulation of the DCF drug into the pore walls, again supporting the accomplishment of premises for a sustained release of the drug.

**Figure 4:**
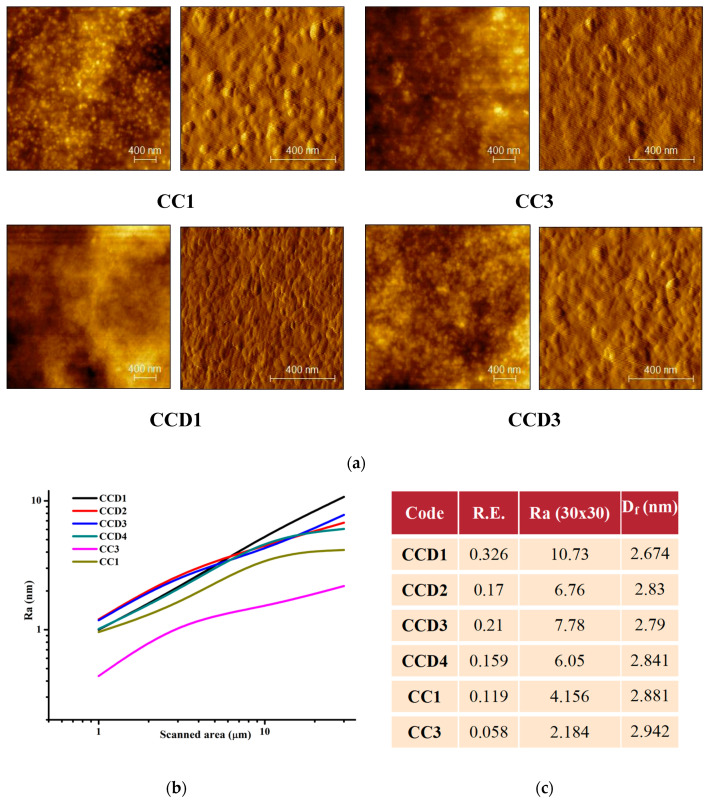
(**a**) Atomic force microscopy (AFM) images of **CCD1** and **CCD3** drug delivery systems and **CC1** and **CC3** reference xerogels; (**b**) graphical representation of roughness vs. scan size; and (**c**) the obtained values for the roughness exponent (R.E.), average roughness (Ra), and fractal dimension (Df) for the **CCD1-CCD4** drug delivery systems and two reference xerogels **CC1** and **CC3**.

In order to evaluate the morphology of both drug delivery systems and the reference hydrogels at a nanometric level, the samples were analyzed by atomic force microscopy in topographic mode, beginning with a scan size of 30 × 30 and decreasing it up to 1 × 1 μm^2^. As could be observed, all of the samples presented a granular morphology with nanometric grains in the range of 35 and 50 nm, indicating nanostructured surfaces. Representative AFM images are presented in Figure 4. For obtaining a deeper insight into the topography of the drug delivery systems, the roughness exponent was calculated as the slope of roughness vs. the scan size in a double logarithmic plot [30,31]. The obtained values of the roughness exponent were used to determine the fractals dimensions according to the following mathematical equation:

*D_f_* = 3 − *R.E.* (3)

Therefore, all of the investigated samples, reference xerogels, and drug delivery systems presented values of the fractal dimension close to 3, revealing that the surface of the samples looks more like a volume than a bidimensional surface. Moreover, recent studies demonstrated that the proliferation rate of fibroblasts and mesenchymal stem cells on hydrogels grows directly proportionally to the fractal dimension, indicating that these hydrogels are also promising for tissue engineering [32].

On the other hand, differences in terms of phase contrast shift values were observed between the reference hydrogels and the drug-containing ones. The reference xerogels presented low values of the phase contrast shift, being between 30 and 40 nm, while the drug delivery systems presented much higher values of the same parameter, being between 60 and 125 nm. This clearly indicated relief differences in the case of the reference xerogels, revealing the chemical homogeneity of the systems and chemical composition variation in the case of the drug delivery systems, due to the drug encapsulation and segregation into the hydrogel matrix at a nanometric scale [33].

### 3.4. In Vitro Enzymatic Degradation in the Presence of Lysozyme

**Figure 5:**
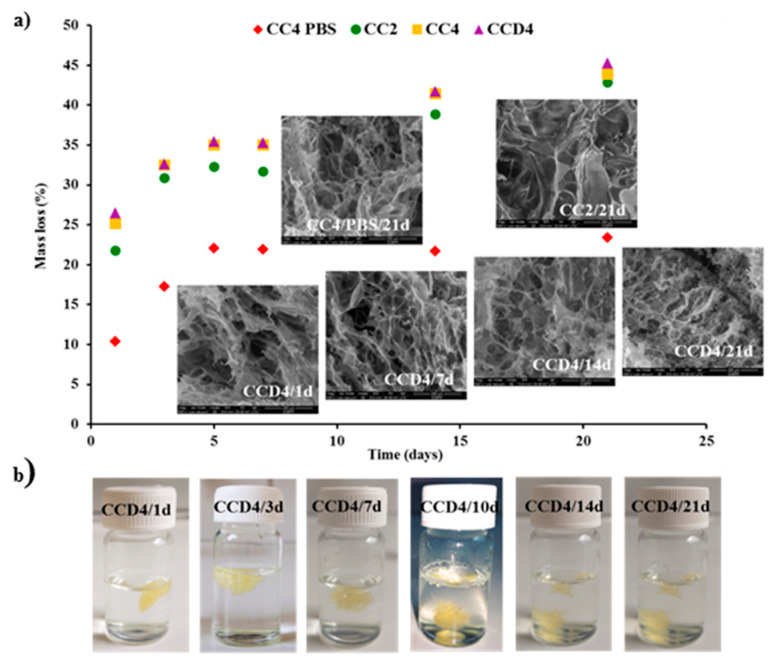
(**a**) Graphical representation of mass loss vs. time and several representative SEM images, and (**b**) images of the **CCD4** drug delivery system during the enzymatic degradation experiment.

Biodegradability under enzymatic conditions is an important demand for biomaterials [34]. Therefore, we investigated the degradation of citryl-imino-chitosan xerogels and drug delivery systems in the presence of lysozyme. For comparison, we also evaluated the degradability in the presence of PBS. The mass loss of the samples and the morphological changes, due to the hydrolytic and/or enzymatic degradability, were evaluated gravimetrically and by SEM, respectively (Figure 5).

No significant differences were noted between the values of the mass loss in the case of the drug delivery systems in comparison to the reference xerogels, revealing that the DCF drug encapsulated into the matrix did not influence the enzymatic degradability of the xerogels. Interesting enough, quite high values were obtained for the mass loss, and were much higher than expected for a chitosan with such a high deacetylation degree. This clearly indicates that, along with the enzymatic degradation of the matrix due to lysozyme, which involves the rupture of C-O-C linkages between two N-acetylglucosamine units, hydrolytic degradation occurs due to the presence of reversible imine linkages. Moreover, physical ageing of chitosan leading to chitosan oligomers with an improved solubility by chain scissions should not be neglected [35,36]. The impact of such processes was confirmed by the quite important mass loss for the neat xerogels when maintained in PBS, ~22% after 21 days, in comparison to 42% in lysozyme’s presence. It can be envisaged that the enzymatic and hydrolytic degradation are competing phenomena, influencing each other towards progressive matrix erosion.

Looking at the evolution of the xerogels in the investigation of the enzymatic biodegradation (Figure 5), it can be seen that xerogel swelling took place slowly, with the xerogels’ density becoming equal to/higher than that of water after 10 days, when more than 30% mass loss was reached.

To obtain a visual insight into the morphological changes due to the hydrolytic or/and enzymatic degradation, SEM was performed on the lyophilized samples. As could be observed, the samples presented smaller or larger cracks in the xerogel pore walls, which was more evident in the case of the samples which suffered from enzymatic degradation and especially after 21 days.

Therefore, both the gravimetric measurements and the morphological investigations revealed the biodegradability of the drug delivery systems and reference hydrogels, making them suitable for in vivo bioapplications.

### 3.5. In Vitro Drug Release

**Figure 6:**
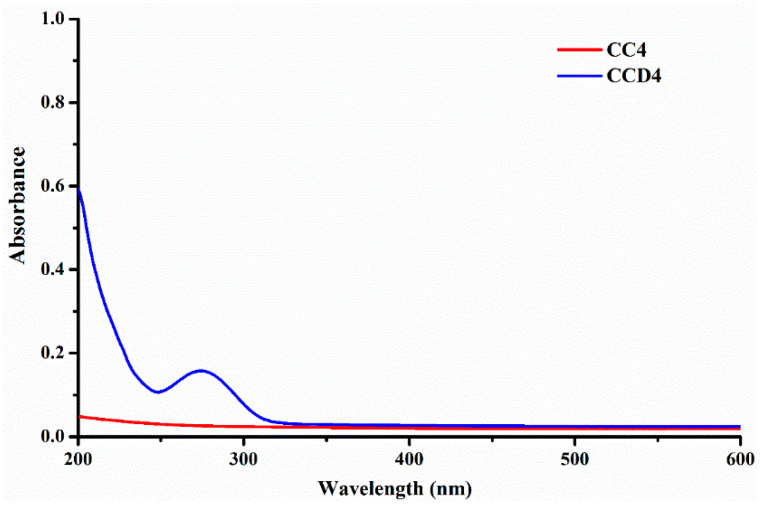
UV-VIS spectrum for **CC4** and **CCD4** samples dissolved in PBS (1/9 *v*/*v*).

In order to investigate the success of the theoretical design according to which the use of an anionic model drug—diclofenac sodium salt—in combination with the positively charged chitosan, should lead to drug delivery systems with sustained release, the release kinetics was monitored in PBS, at 37 °C. At first, we evaluated the possibility to monitor the release kinetics by UV-VIS spectroscopy, by comparing the spectrum of a reference sample with that of the corresponding drug delivery system. As it could be observed, the **CC4** reference hydrogel did not present any absorbance, while the **CCD4** drug delivery system only presented the maximum characteristic of the DCF drug (Figure 6). This data clearly indicated that **DCF** release from the designed drug delivery systems can be monitored by UV-VIS spectroscopy.

**Figure 7:**
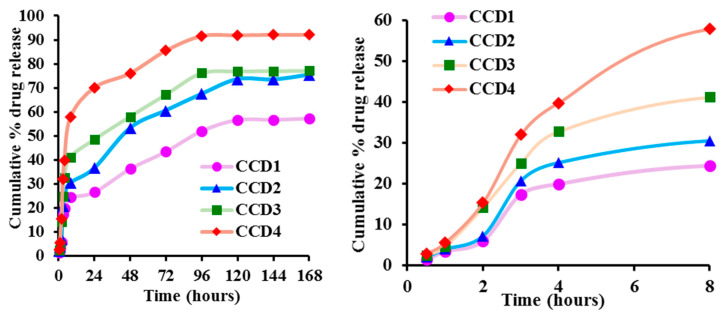
Drug release profiles for the **CCD1-CCD4** drug delivery systems (**a**) for 7 days and (**b**) 8 h. The values are presented as the mean of three individual measurements ± S.D.

Therefore, all of the samples released the encapsulated drug in a sustained manner, in two stages: A first one which lasted up to 4 h, in which a burst release was observed, and a second one, lasting up to 7 days, in which slower release rates were obtained, in line with the slower concentration of the drug in the polymeric matrix. The drug release rate mainly depends on the density of the hydrophobic imine clusters, decreasing with the increase of their number and density (Figure 7, Table 3). Therefore, the sample **CCD4**, which presents the loosest network and therefore the highest elasticity, released the encapsulated DCF drug with a faster rate than the other samples, and in only four hours, released up to 40% of the entire amount of the encapsulated drug. On the other hand, the sample **CCD1**, which has a more robust and compact network, with a higher density of hydrophobic clusters, was able to release the encapsulated drug with a lower rate, and in the same time interval, only released 20% of the amount of the encapsulated DCF. Even if the samples presented different release rates, in all cases, the same release profile was observed, characteristic of sustained release drug delivery systems. This can be explained if it is taken into consideration that, even if the samples were obtained by varying the molar ratio between chitosan and citral, their intrinsic chemical composition was the same. This being said, it can be concluded that both the presence of the polycationic chitosan and the use of the anionic DCF drug can generate strong electrostatic interactions which keep the drug in the polymeric matrix for a longer period of time. Moreover, the existence of numerous hydroxyl groups in chitosan’s structure, along with the presence of two chlorine atoms and an esteric group in DCF’s structure, can generate hydrogen bonds, making DCF release even more difficult.

Therefore, the achievement of different release rates, mainly depending on the number and density of hydrophobic clusters, is the basis of the versatility of these systems, making them suitable for a large range of applications in various diseases, with different therapeutic approaches, when a slow release is required, or in acute diseases in which a shock dose is necessary.

### 3.6. The Analysis of Drug Release Kinetics

All of the data obtained up to now revealed that the understudy drug delivery systems are characterized by a uniform drug distribution and offer a sustained release due to the strong interactions established between the DCF drug and the polymeric matrix. In order to obtain a deeper insight into the release process and to better understand the factors which govern it, the kinetic data were fitted with some mathematical models, including zero order, first order, Higuchi, Korsmeyer–Peppas, and Hixson–Crowell models. The fitting was done separately in two stages: 0–4 h and 8–96 h.

Looking at the correlation coefficients obtained for the fitting of the first stage kinetic data, it could be observed that the mechanism of release of the DCF drug from the matrix depends on the intrinsic chemical structure of the matrix and more precisely, on the density and number of hydrophobic clusters. Therefore, in the case of the **CCD3** and **CCD4** hydrogels, which present the loosest networks, the dependence between the % drug released and time, according to the zero order model, is linear, with a correlation coefficient higher than 0.99. This clearly indicated drug dissolution as an important factor which influences DCF release from the two hydrogels. In the case of the **CCD1** and **CCD2** samples, characterized by a higher density of hydrophobic clusters and lower ability to swell, the zero order kinetic model led to low values of the correlation coefficient (Table 4) [37,38]. Moreover, in the case of the first two samples mentioned before, the fitting of the kinetic data with the first order model indicated the dependency between the amounts of encapsulated and respectively, released drug. The values of the K_0_ and K_t_ constants increased from the sample **CCD1** to **CCD4**, with the increase of the matrix hydrophilicity, presenting a good correlation with the rate of rehydration. The Higuchi model did not fit very well in the first stage, showing that the diffusion of the drug through the polymeric matrix is not the main process which influences the drug release [39]. On the other hand, the fitting of the Hixson–Crowell model to the kinetic data led to high values of the correlation coefficient in the case of the **CCD3** and **CCD4** samples, again showing that, in these cases, DCF dissolution is more important than drug diffusion. The values of the n_r_ parameter obtained in the case of the Korsmeyer–Peppas model, which are higher than 1, indicated non-Fickian supercase II diffusion and showed that DCF release from the hydrogels is a complex process in which many factors play a role, such as matrix swelling and erosion, on one hand, and drug diffusion and dissolution, on the other hand [40,41].

By analyzing all these data, it could be observed that the zero order and first order models led to the highest values for the correlation coefficient, especially in the case of two samples, revealing the drug dissolution as the main factor which influences DCF release in the first 4 h of the releasing process.

The use of the same mathematical models for the kinetic data in the second stage led to higher values of the correlation coefficients in almost all cases (except for the Korsmeyer–Peppas model for sample **CCD1**), revealing that the mechanism of release changes over time and turns into a more complex one in which many factors play an equally important role, being no longer possible to highlight the crucial one (Table 5). The n_r_ values obtained when the Korsmeyer–Peppas model was used were significantly lower than the ones obtained in the first stage, indicating that the diffusion process changed from a non-Fickian to Fickian one [39,40,41].

## 4. Conclusions

This study presents the synthesis of novel drug delivery systems based on biocompatible citryl-imine-chitosan hydrogels. The diclofenac sodium salt, used as an anionic drug model, was encapsulated simultaneously with chitosan’s hydrogelation process, which allowed the homogeneous entrapment of the drug into the pores’ walls, at a nanometric scale, as was indicated by SEM, POM, and AFM images. The monitoring of DCF delivery from the novel drug delivery systems revealed a sustained release profile, correlated with the strong interactions developed between the anionic DCF molecules and cationic citryl-imine-chitosan matrix. Moreover, the enzymatic degradability tests showed that these materials are highly biodegradable, reaching a maximum of 42% erosion in the presence of lysozyme. All these data confirm that citryl-imine-chitosan hydrogels are promising materials for bioapplication, presenting properties which recommend them for the development of efficient drug delivery systems in terms of drug encapsulation and the drug release rate, representing characteristics which can be tuned by the ratio between the hydrophobic and hydrophilic parts.

## Figures and Tables

**Table 1:** The composition of the drug delivery systems (**CCD1-CCD4**) and the reference hydrogels (**CC1-CC4**).

	Sample Code	m _chitosan_ (mg)	V _water_ (mL)	V _acetic acid_ (μL)	m _citral_ (mg)	m _DCF_ (mg)	V _ethanol_ (mL)	m_1_^1^ (mg)	m_2_^2^ (mg)	η (%)
**Drug delivery systems**	**CCD1**	33.89	1.129	11.85	26.606	1.5	2.81	62	61.8	99.7
	**CCD2**	43.47	1.449	15.214	17.02	1.5	1.852	62	61.9	99.8
	**CCD3**	47.95	1.598	16.78	12.54	1.5	1.404	62	61.9	99.8
	**CCD4**	50.62	1.687	17.71	9.87	1.5	1.137	62	61.8	99.7
**References samples**	**CC1**	33.89	1.129	11.85	26.606	-	2.606	60.5	60.3	99.66
	**CC2**	43.47	1.449	15.214	17.02	-	1.702	60.5	60.2	99.5
	**CC3**	47.95	1.598	16.78	12.54	-	1.254	60.5	60.2	99.5
	**CC4**	50.62	1.687	17.71	9.87	-	0.987	60.5	60.2	99.5

^1^ m_1 =_ the amount of all reactants in a dried state added to the reaction; ^2^ m_2_ = the xerogel mass, weighted after sample lyophilization; and η = the reaction yield.

**Table 2:** Morphological parameters for the drug delivery systems and reference samples.

Sample	Mean Diameter of Pores (μm)	Standard Deviation	Wall Thickness of Pores (μm)
**CCD1**	9	2.2	5
**CCD2**	9.2	1.1	5
**CCD3**	15.7	4	10
**CCD4**	22	3.2	10
**CC1**	10.8	1.09	2
**CC2**	10	1.34	3
**CC3**	20.6	1.94	5
**CC4**	27	2.1	5

**Table 3:** Standard deviation values for the analyzed samples obtained for DCF release kinetics.

Time (h)	S.D. CCD4	S.D. CCD3	S.D. CCD2	S.D. CCD1
0.5	0.6856	0.0058	0.0041	0.0005
1	1.1953	0.0022	0.0012	0.0069
2	3.3243	0.0054	0.0057	0.0057
3	4.0099	0.0105	0.0082	0.0125
4	5.0722	0.0024	0.0050	0.0120
8	8.4162	0.0068	0.0083	0.0069
24	2.9715	0.0107	0.0119	0.0024
48	3.8146	0.0094	0.0022	0.0017
72	5.3151	0.0086	0.0164	0.0221
96	1.4805	0.0024	0.0056	0.0014
120	1.2566	0.0168	0.0086	0.0098
144	1.0607	0.0024	0.0064	0.0039
168	0.0667	0.0036	0.0129	0.0051

**Table 4:** The kinetic data obtained in the first stage of 0–4 h.

Code	Zero Order		First Order		Higuchi Model		Hixson–Crowell		Korsmeyer–Peppas		
	R^2^	K_0_	R^2^	K_t_	R^2^	K_H_	R^2^	Κ	R^2^	k	n_r_
**CCD1**	0.94	5.71	0.94	0.046	0.9	15.25	0.94	−0.095	0.97	0.3	1.29
**CCD2**	0.94	7.08	0.94	0.069	0.9	18.82	0.94	−0.011	0.97	0.23	1.24
**CCD3**	0.99	9.06	0.99	0.11	0.97	21.35	0.99	−0.159	0.99	0.18	1.31
**CCD4**	0.98	11.23	0.98	0.13	0.95	30.09	0.98	−0.205	0.99	0.15	1.33

R^2^: correlation coefficient; K: proportionality constant; and n: release exponent.

**Table 5:** The kinetic data obtained in the second stage of 8–96 h.

Code	Zero Order		First Order		Higuchi Model		Hixson–Crowell		Korsmeyer–Peppas		
	R^2^	K_0_	R^2^	K_t_	R^2^	K_H_	R^2^	Κ	R^2^	k	n_r_
**CCD1**	0.99	0.32	0.98	0.004	0.95	4.048	0.99	−0.006	0.89	0.085	0.305
**CCD2**	0.97	0.43	0.99	0.007	0.98	5.58	0.98	−0.108	0.96	0.07	0.33
**CCD3**	0.99	0.39	0.99	0.005	0.98	5	0.99	−0.011	0.96	0.042	0.24
**CCD4**	0.96	0.36	0.98	0.016	0.99	4.72	0.99	−0.015	0.98	0.025	0.17

R^2^: correlation coefficient; K: proportionality constant; and n: release exponent.
